# Preconception care practice among pregnant women attending antenatal care at Wachemo University, Nigist Eleni Mohammed Memorial Comprehensive Specialized Hospital, southern Ethiopia, 2022: a mixed-methods study

**DOI:** 10.3389/fgwh.2025.1569410

**Published:** 2025-09-04

**Authors:** Tadesse Getu, Melesech Eliso, Mengistu Lodebo, Tesema Beraku, Temesgen Getaneh, Engdaw Asmare Anlay, Samuel Yohannes, Pammla Petrucka, Ayenew Tega

**Affiliations:** 1 Department of Midwifery, Hosanna Health Science College, Hosanna, Ethiopia; 2 Department of Midwifery, College of Medicine and Health Science, Debre Markos University, Debre Markos, Ethiopia; 3 College of Nursing, University of Saskatchewan, Saskatoon, SK, Canada

**Keywords:** preconception care, practice, pregnant women, southern Ethiopia, mixed-methods study

## Abstract

**Background:**

Preconception care includes biomedical, behavioral, and social health interventions for women and couples before conception, aiming to improve their overall health status. Despite its importance, studies conducted in Ethiopia reveal that the practice remains unacceptably low; this emphasizes the need for further investigation, particularly through mixed-methods studies incorporating women's perspectives.

**Methods:**

An institution-based, cross-sectional study with concurrent triangulation was conducted at Wachemo University, Nigist Eleni Mohammed Memorial Comprehensive Specialized Hospital between 1 April and 30 June 2022. Quantitative data were collected using a systematic random sampling method, while qualitative data were obtained through purposive sampling. A structured, interviewer-administered questionnaire was used to collect data from 332 eligible antenatal care clients. The data were entered into EpiData and analyzed using SPSS. Bivariate and multivariate analyses were performed to identify factors associated with the practice of preconception care. A 95% confidence interval (CI) and *p*-values <0.05 were considered statistically significant. Thematic analysis of qualitative data was performed using ATLAS.ti version 7.

**Results:**

This study showed that 104 women (31.3%) (95% CI: 26.5–36.5) engaged in good preconception care practices. Factors significantly associated with good practices included attending college or university [adjusted odds ratio (AOR) = 3.52, 95% CI: 1.14–10.87], having a history of adverse pregnancy outcomes (AOR = 4.82, 95% CI: 2.20–10.58), receiving support from one’s husband (AOR = 2.45, 95% CI: 1.05–5.73), and having good knowledge of preconception care (AOR = 4.52, 95% CI: 2.11–9.68). The qualitative analysis revealed that client-related and health facility-related factors influenced the practice of preconception care.

**Conclusion:**

Nearly 7 out of 10 women in this study became pregnant without the utilization of any component of preconception care. To improve the practice of preconception care, it is essential to raise awareness about its benefits among all women of reproductive age. Future efforts focusing on knowledge dissemination and awareness creation to improve partner support are crucial to enhancing preconception care practices.

## Introduction

Preconception care (PCC) refers to the provision of biomedical, behavioral, and social health interventions to women and couples before conception to improve health status and minimize risk factors that may contribute to poor maternal and child health outcomes (1). PCC addresses the health of women and their partners before pregnancy and is recognized as an essential element for achieving healthy outcomes for mothers and their children (2). PCC aims to identify and, when possible, modify risk factors that are directly or indirectly linked to untoward perinatal outcomes (3).

PCC contributes to reducing maternal and childhood mortality and morbidity in addition to improving maternal and child health, in both high- and low-income countries by preventing or reducing anemia, unplanned pregnancies, sexually transmitted diseases, adolescent pregnancies, congenital anomalies, stillbirths, low birth weight, and preterm births (1, 4). PCC potentially averts 71% of neonatal deaths, 33% of stillbirths, and 54% of maternal deaths per year (5). In addition, it accelerates the seeking of antenatal care (ANC) by 39% and positively influences the continuum of maternal care (6). Due to this effect, Ethiopia has set target goals to increase the number of women who receive PCC by 25% to reduce maternal and neonatal deaths (7).

Despite its recognized importance, evidence shows that uptake of PCC among women of reproductive age remains inadequate. According to the World Health Organization, globally, 4 out of 10 women report that their pregnancies were unintended. As a result, 40% of pregnancies do not receive the necessary healthcare services before pregnancy (8). Evidence shows that PCC utilization is low in many countries, with rates of 40% in China (9), 15.9% in Brazil (10), 47% in Iran (11), 24.05% in sub-Saharan Africa (12), and 16.27% in Ethiopia (13). This limited implementation of PCC significantly contributes to maternal and childhood mortality and morbidity (1). For example, the Ethiopian Demographic Health Survey reported that 58% of women did not take iron or folate tablets during their most recent pregnancy (10), despite strong evidence that folate supplementation reduces the risk of neural tube defects (14).

Findings from different literature have shown that sociodemographic factors, such as age, marital status, residence, educational status, spousal support, family income, good knowledge of PCC, along with obstetrical factors such as multiparity, previous adverse pregnancy outcomes, and history of chronic illness, were associated with PCC practices (15–19).

Although various studies have been conducted in Ethiopia to assess PCC practices, the majority do not address women's views on PCC. Therefore, this study aimed to assess the level of practice, identify associated factors, and explore women's views on PCC among pregnant women attending Nigist Eleni Mohammed Memorial Comprehensive Specialized Hospital, Southern Ethiopia, using a mixed-methods approach.

## Methods

### Study design and setting

An institutional-based, quantitative, cross-sectional study with concurrent triangulation with qualitative interview findings was conducted between 1 April and 30 June 2022. The study was conducted at Wachemo University, Nigist Eleni Mohamed Memorial Comprehensive Specialized Hospital, located in Hosanna Town, Southern Ethiopia. It is the only comprehensive specialized hospital found in the Hadiya Zone. Hosanna Town is situated 232 km from Addis Ababa, the capital city of Ethiopia. The hospital was established in 1976 and serves approximately 1,568,000 men and 1,632,000 women annually.

### Study population

All pregnant women attending ANC at Wachemo University, Nigist Eleni Mohammed Memorial Comprehensive Specialized Hospital during the data collection period were included in the study, and purposively selected pregnant women were included for the qualitative component. However, women who were severely ill, specifically those unable to communicate, were excluded from participation.

### Sample size and sampling procedure

The sample size was determined using the single population proportion formula by considering the following assumptions: confidence interval (CI) = 95%; critical value (Z*α*/2)^2^ = 1.96; and degree of precision d = 0.04. The proportion (p) = 14.5% was obtained from a study conducted in West Shewa, Oromia, Ethiopia (16). This resulted in a sample size of 332 pregnant women, including a 10% non-response rate. The data collection period for this study was 3 months. A total of 747 pregnant women received ANC services in the 3 months preceding data collection. Based on this, the sampling interval was determined by dividing the total number of 747 by a calculated sample size of 332, resulting in a sampling interval of approximately 2. Finally, every other ANC patient was selected for an interview within 3 months. The first pregnant woman was randomly selected from among the first two based on the sequence of their charts.

For the qualitative component, the purposive sampling technique was used to select participants until data saturation was achieved, which occurred with eight participants. Qualitative interview participants were selected from those not involved in the quantitative component.

### Study variables

The practice of PCC among pregnant women was the dependent variable and included receiving at least one of the “good practice” components of PCC [i.e., folic acid supplementation, immunization, avoiding cigarette smoking, avoiding illegal drugs, maintaining a balanced diet, caring for one’s psychosocial health, screening for sexually transmitted infections (STIs) and human immunodeficiency virus (HIV), avoiding environmental hazards, exercising regularly, and receiving early medical checkups] (20). This threshold was chosen based on the limited integration of PCC into routine maternal health services in the study setting, as comprehensive PCC is not routinely available in this context. In the absence of comprehensive and fully integrated PCC services, receiving even a single PCC component was considered a meaningful indicator of good practice. Given these contextual limitations, such minimal engagement reflects both awareness of and initial contact with preconception services, which is particularly significant in low-resource settings. However, those who did not engage in any PCC component in preparation for pregnancy were categorized as having poor PCC practice (20).

Maternal socioeconomic, demographic, obstetric, and medical-related traits, along with access to health facilities and knowledge-related variables, were considered independent variables.

Knowledge of PCC was assessed by 12 yes-or-no questions. Study participants who answered ≥50% of the knowledge questions correctly (17).

### Data collection tool and procedure

The data collection tool was adapted after reviewing different related articles and documents (16–19). First, the tool was prepared in English and then translated into Amharic. Maternal socioeconomic and demographic data, obstetric and medical-related data, access to health facility-related data, knowledge, and practice-related data were included in the tool. The data were collected using structured and pre-tested interviewer-administered questionnaires. Six midwives with bachelor's degrees were recruited as data collectors for the quantitative part, and two experienced supervisors were assigned to oversee the data collection process and to check the quantitative data for completeness, accuracy, and clarity. Qualitative data were collected by the principal investigator through audio-recorded interviews using semi-structured questions. The in-depth, individual interviews took place in a quiet room suitable for audio recording. Data collection and participant probing continued until thematic saturation was reached.

### Data quality assurance

The questionnaires were pre-tested at Worabe Comprehensive Specialized Hospital on 5% of the sample size of pregnant women, 1 week before the actual data collection period. A 2-day training was given to the data collectors and supervisors. Two assigned supervisors provided close supervision throughout the entire quantitative data collection period to check data for completeness, accuracy, and clarity. The interviews were conducted privately, with assurances of confidentiality to minimize social desirability bias. Furthermore, the collected data were entered into EpiData version 3.1 to minimize data entry errors. The reliability of the practice assessment tool was evaluated using Cronbach's alpha, which resulted in a value of 0.72, indicating acceptable internal consistency. The qualitative data were transcribed, and the principal investigator checked the consistency of the transcription.

### Data processing and analysis

The collected data were entered into EpiData version 3.1 and exported to SPSS version 23 for cleaning, coding, computing, and analysis. All explanatory variables with a *p*-value <0.25 in the bivariable logistic regression analysis were candidate variables for the multivariable logistic regression analysis. Finally, after adjusting for confounding factors, a *p*-value <0.05 was used to determine the final statistical significance of this study. The principal investigator listened to the audio recordings, transcribed the interviews verbatim, and translated them into English with the assistance of another author (AT). The translated data were read and reread until the full meaning of the contents was understood and familiar to the data. The translated data were coded using ATLAS.ti version 7 qualitative analysis software; codes with similar meanings were then grouped into subcategories and main themes for thematic analysis.

### Ethics approval and consent to participate

Ethical clearance was obtained from the Institutional Review Board (IRB) of the Hosanna College of Health Sciences (Ref. No. Code 1102/2013 E.C.). After communicating with the responsible hospital managers, permission letters were obtained. Before data collection, written informed consent was obtained from quantitative and qualitative study participants.

## Results

### Sociodemographic characteristics

A total of 332 pregnant women participated, resulting in a response rate of 100%. The ages of the participants ranged from 15 to 45 years (mean age 25.7 ± 4.3 years). The majority (84%) of the study participants were of the Hadiya ethnicity, and nearly three-quarters (74.4%) were Protestant. Nearly one-fifth (21.7%) of the respondents had no formal education, and one-half (50.3%) were housewives (Table 1).

**Table 1:** Sociodemographic characteristics of the study participants at Wachemo University, Nigist Eleni Mohammed Memorial Comprehensive Specialized Hospital, southern Ethiopia, 2022 (*n* = 332).

Variables	Response	Frequency	Percentage
Residency	Urban	296	89.2
	Rural	36	10.8
Age	15–24	132	39.8
	25–34	186	56.0
	35–49	14	4.2
Marital status	Single	8	2.4
	Married	324	97.6
Ethnicity	Hadiya	279	84.0
	Kenbata	15	4.5
	Gurage	18	5.4
	Sltie	7	2.1
	Amhara	13	3.9
Religion	Protestant	253	74.4
	Orthodox	50	15.1
	Muslim	27	8.1
	Catholic	8	2.4
Educational level	No formal education	72	21.7
	Primary education	96	28.9
	Secondary education	96	28.9
	College/university	68	20.5
Occupation	Student	34	10.2
	Government employee	73	22.0
	Private business	58	17.5
	Housewife	167	50.3
Husband’s educational level	No formal education	10	3.0
	Primary education	87	26.2
	Secondary education	118	35.5
	College/university	117	35.2
Occupation of husband	Student	12	3.6
	Government employee	110	33.1
	Private business	153	46.1
	Daily labor	14	4.2
	Farmer	43	13.0
Family size	<4	225	67.8
	≥4	107	32.2
Monthly income	<1,001	26	7.8
	1,001–3,000	84	25.3
	3,001–5,000	81	24.4
	>5,000	141	42.5

### Obstetrical and pre-existing medical conditions of the study participants

Of the total study participants, 137 (41.3%) women were pregnant for the first time, and 102 (52.3%) respondents were multiparous. A total of 61 participants (31.3%) reported a history of adverse pregnancy outcomes, including abortion (49%) and stillbirth (19.7%). Among the total participants, 17 (5.1%) had a pre-existing medical condition (Table 2).

**Table 2:** Obstetrical and medical-related characteristics of the study participants at Wachemo University, Nigist Eleni Mohammed Memorial Comprehensive Specialized Hospital, southern Ethiopia, 2022 (*n* = 332).

Variables	Response	Frequency	Percentage
Number of pregnancies/gravidities	Primigravida	137	41.3
	Multigravida	195	58.7
History of antenatal care visits for previous pregnancies	Yes	173	88.7
	No	22	11.3
Number of deliveries (*n* = 195)	Nulliparous	9	4.6
	Primiparous	84	43.1
	Multiparous	102	52.3
Place of delivery for the last birth (*n* = 186)	Health facility	180	93.6
	Home	12	6.4
Mode of delivery for the last birth (*n* = 186)	Vaginal delivery	158	85
	Cesarean section	28	15
History of adverse pregnancy outcomes (*n* = 195)	Yes	61	31.3
	No	134	68.7
Type of adverse pregnancy outcome (*n* = 61)	Abortion	30	49
	Stillbirth	12	19.7
	Preterm birth	10	16.5
	Congenital anomaly	4	6.6
	Preeclampsia	5	8.2
History of previous postnatal care services	Yes	80	43
	No	106	57
History of utilization of family planning services	Yes	191	57.5
	No	141	42.5
Pre-existing medical condition	Yes	17	5.1
	No	315	94.9
Type of pre-existing medical condition (*n* = 17), multiple responses are possible	HIV/AIDS	4	18.2
	DM	6	27.3
	Hypertension	7	31.8
	Anemia	5	22.7

HIV/AIDS, Human immunodeficiency virus/acquired immunodeficiency syndrome; DM, diabetes mellitus.

### Access to health facility-related characteristics

Among the total study participants, 240 (72.3%) perceived that the distance to reach a nearby health facility on foot was less than 30 min. The majority of respondents (*n* = 281, 84.6%) had access to transportation, and 302 (90.9%) reported that medications were available. More than two-thirds (*n* = 227, 68.3%) of the study participants received support from their husbands for health services, and 242 (72.9%) had autonomy in making decisions about maternal health services.

### Level of knowledge of study participants

Three-fifths (58.3%) of the study participants had heard about PCC. The main sources of information were family and relatives (28.6%), healthcare providers (19.3%), and schools (9.6%).

Regarding knowledge of practices contributing to a healthy pregnancy outcome, 204 participants (61.4%) recognized the importance of folic acid supplementation, 160 women (48.2%) recognized the importance of vaccinations, 270 respondents (81.3%) recognized the importance of exercise, and 172 women (51.8%) recognized the importance of maintaining an optimal body weight. In addition, participants reported the following as important pre-pregnancy practices: screening for chronic conditions (58.1%), testing for STIs (79.5%), avoiding alcohol consumption (46.4%), refraining from illegal drug use (58.4%), screening for psychosocial problems (46.4%), and avoiding exposure to environmental hazards (34.9%). Overall, the study showed that fewer than half of the participants (*n* = 158, 47.6%; 95% CI: 42.3–53) had good knowledge of PCC.

### The level of practice of preconception care among study participants

This study showed that 104 (31.3%) of the participating women (95% CI: 26.5–36.5) observed good PCC practices. Furthermore, 7 in 10 women were currently pregnant without receiving PCC. Fewer than one-quarter (23.2%) of the women reported taking folic acid, and fewer than one-fifth received counseling on a balanced diet before their current pregnancy (Table 3). Qualitative findings supported these results, revealing that the majority of women did not practice PCC.

**Table 3:** Level of preconception care practices among the study participants at Wachemo University, Nigist Eleni Mohammed Memorial Comprehensive Specialized Hospital, southern Ethiopia, 2022 (*n* = 332).

Variables	Response	Frequency	Percentage
Folic acid supplementation before pregnancy	Yes	77	23.2
	No	255	76.8
Immunization before pregnancy	Yes	27	8.1
	No	305	91.9
Counseled on avoiding cigarette smoking	Yes	93	28.0
	No	239	72.0
Counseled on avoiding taking illegal drugs	Yes	85	25.6
	No	247	74.4
Advice on diet/nutrition before pregnancy	Yes	62	18.7
	No	270	81.3
Received psychosocial counseling before pregnancy	Yes	56	16.9
	No	276	83.1
Received screening, counseling, and testing services for STIs and HIV before pregnancy^a^	Yes	67	20.2
	No	265	79.8
Received counseling to avoid environmental hazards before pregnancy	Yes	73	22.0
	No	259	78.0
Practicing regular exercise before pregnancy	Yes	67	20.2
	No	265	79.8
Attending the hospital for an early medical checkup before pregnancy	Yes	60	18.1
	No	272	81.9

STIs, sexually transmitted infections; HIV, human immunodeficiency virus.

“I am neither infertile nor ill, so I do not feel the need to visit a health facility before conception. Women seek healthcare before pregnancy only when they are ill or experiencing infertility” (34-year-old multigravida housewife).

### Factors associated with the practice of preconception care

Bivariate and multivariable logistic regression analyses were conducted to identify factors affecting the practice of PCC. In the bivariable analysis, educational status, history of adverse pregnancy outcomes, planned status of the current pregnancy, decision making autonomy regarding maternal health service, receiving support from a spouse, distance from the nearest health facility, and knowledge of PCC were candidate variables for the multivariable logistic regression analysis (*p*-value <0.25). After controlling for the confounding factors, the multivariable analysis revealed that educational status, history of adverse pregnancy outcomes, receiving support from a spouse, and knowledge of PCC were identified as being significant (*p*-value <0.05).

This study showed that women who attended college or university were 3.52 times more likely to observe good PCC practices than participants without a formal education [adjusted odds ratio (AOR) = 3.52, 95% CI: 1.14–10.87].

Participants who had a history of adverse pregnancy outcomes were 4.82 times more likely to observe good PCC practices compared to their counterparts (AOR = 4.82, 95% CI: 2.20–10.58). This finding is supported by qualitative results, as women who had a history of adverse pregnancy outcomes were counseled to have follow-ups before future conception to prevent the recurrence of the problem.

“My neighborhood woman, who had a history of giving birth to anencephaly, was counseled to take folic acid 3 months before pregnancy” (29-year-old, multigravida, housewife).

Participants who received support from their husbands were 2.45 times more likely to observe good PCC practices compared to their counterparts (AOR = 2.45, 95% CI: 1.05–5.73).

The present study found that women with good knowledge of PCC were 4.52 times more likely to practice it than those with poor knowledge (AOR = 4.52, 95% CI: 2.11–9.68) (Table 4). In addition, qualitative studies identified the challenge of sourcing reliable and easily available preconception health information as a barrier to utilizing the service.

**Table 4:** Bivariable and multivariable logistic regression analysis of factors associated with preconception care practices among pregnant women at Wachemo University Nigist Eleni Mohammed Memorial Comprehensive Specialized Hospital, southern Ethiopia, 2022 (*n* = 332).

Variables	Preconception care services		COR (95% CI)	AOR (95% CI)	*p*-value
	Good	Poor			
Educational status					
No formal education	15 (20.8%)	57 (79.2%)	1	1	
Primary	25 (26%)	71 (74%)	1.33 (0.65–2.77)	1.09 (0.37–3.17)	0.99
Secondary	25 (26%)	71 (74%)	1.33 (0.65–2.77)	1.51 (0.49–4.65)	0.38
College and above	29 (42.6%)	39 (57.4%)	5.11 (2.43–10.76)	3.52 (1.14–10.87)	0.03*
Presence of previous adverse pregnancy outcomes					
Yes	33 (54.1%)	28 (45.9%)	4.27 (2.23–8.18)	4.82 (2.20–10.58)	0.001**
No	29 (21.6%)	105 (78.4%)	1	1	
Was the current pregnancy planned?					
Yes	96 (33.1%)	194 (66.9%)	2.10 (0.94–4.72)	0.78 (.024–2.51)	0.78
No	8 (19%)	34 (81%)	1	1	
Decision making autonomy regarding maternal health service					
Yes	86 (35.5%)	156 (64.5%)	2.21 (1.24–3.94)	0.54 (0.19–1.57)	0.53
No	18 (20%)	72 (80%)	1	1	
Receiving support from husband					
Yes	89 (39.2%)	138 (60.8%)	3.87 (2.11–7.11)	2.45 (1.05–5.73)	0.03*
No	15 (14.3%)	90 (85.7%)	1	1	
Distance from the nearest health facility (min)					
<30	69 (36.1%)	112 (63.9%)	1.71 (1.06–2.78)	1.62 (0.68–3.81)	0.25
≥30	35 (24.8%)	106 (75.2%)	1		
Level of knowledge					
Good knowledge	77 (48.7%)	81 (51.3%)	5.18 (3.09–8.67)	4.52 (2.11–9.68)	0.001**
Poor knowledge	27 (15.5%)	147 (84.5%)	1	1	

COR, crud odd ratio; AOD, adjusted odd ratio.

* Significant at *p*-value <0.05, **statistically significant at *p*-value <0.001.

“Even if I had an ANC follow-up in the previous pregnancy and gave birth in this hospital, no one told me about PCC and the availability of services in this hospital” (32-year-old multigravida housewife).

“I had never heard about the service and was not aware that I could, before being pregnant, go to the clinic and discuss with a healthcare provider my desire for pregnancy. I only visited the clinic for an ANC or when I became ill” (28-year-old multigravida, housewife).

#### Theme 1: client factors

Under this theme, lack of awareness of the service, economic factors, time constraints, and cultural factors were identified.

##### Economic and time constraints

Study participants were not willing to spend time and money on attending PCC. There were costs associated with transportation, laboratory services, and medication, and it was difficult for women who had no income of their own. There are no free services for non-pregnant women; hence, low-income women might be unable to afford them.

“I am a government employee; my vacation was on the weekend, but there is no preconception service on the weekend. There was not enough time to visit health facilities for PCC; the manager does not give me leave” (26-year-old employee primigravida).

“I have no money to pay for transportation service, for investigation, and medication” (34-year-old, housewife, multigravida).

##### Cultural factors

Specifically, women discussed the private and often secretive period between planning a pregnancy and actual conception. Many expressed an unwillingness to share their plans or desires for pregnancy with others, including healthcare professionals. In some communities, there is a common belief that health facilities are intended primarily for treatment rather than prevention, making visits to a healthcare facility seem unnecessary in the absence of apparent health issues.

“I am afraid to talk about my intention to get pregnant, and I have not told my family about being pregnant until three months have passed or until the risk of abortion is ruled out” (23-year-old student, primigravida).

“In my community, when healthy women visit health facilities, they are considered by the community as not liking work” (29-year-old housewife, multigravida).

#### Theme 2: health facility factors

Barriers to the uptake of PCC included a lack of service announcements, the unavailability of PCC at nearby health facilities, long waiting times at clinics, and the cost of services. Study participants reported that even women with illnesses often avoid seeking healthcare due to the high costs associated with investigations and medications. In addition, study participants were uncertain about the presence of a dedicated PCC unit in health facilities, as there were no visible posters or signs providing information or directions to the service.

“PCC services are unavailable at my nearby health facility, even though I was also referred today there for ANC” (28-year-old merchant, multigravida).

“Health facilities have not announced the presence of the service, and health education about PCC was not given to the community, nor was it posted about the service within the facilities” (25-year-old, employee, primigravida).

“Since no free service was given to non-pregnant women, I have no money to pay for PCC services like medication and laboratory. I know that before the current pregnancy, I was sick with malaria, and I paid for the card, laboratory, and medication service provided for me” (34-year-old, housewife, multigravida).

“I am busy, but I stayed a longer time in this hospital to get service, and as a result, I wouldn’t come back again to the hospital for service” (26-year-old employee, primigravida).

## Discussion

The study results showed that one-third (31.3%) of participants (95% CI: 26.5–36.5) followed good PCC practices. This finding is in line with studies conducted in Nigeria (34.1%) (21) and Zambia (33.3%) (22). However, this figure is higher than the findings from studies conducted in Hosanna (19%) (15), Mizan-Aman (16.2%) (23), Fiche (21.6%) (24), West Guji Zone (22.3%) (25), Mekelle (18.2%) (18), and China (20.6%) (26). The higher prevalence in our study may be due to the difference in study settings, as previous studies were conducted in community settings, whereas the present study was conducted at a healthcare facility. Participants at specialized hospitals may have a higher level of healthcare awareness and a higher socioeconomic status, enabling them to understand the importance of PCC and access healthcare services easily. In addition, women attending specialized hospitals may be more likely to be urban-dwellers and present with pre-existing health problems, pregnancy-related complications, or perceived risks, encouraging them to be more proactive in seeking care, including preconception services. Consequently, a study limited to specialized hospital settings potentially overestimates the prevalence of preconception care among the general population of the region.

Conversely, the rate of good PCC practices in this study was lower than that reported in studies from Addis Ababa (40%) (27), Eswatini (59%) (28), Iran (47.7%) (11), Malaysia (45.2%) (29), and China (40%) (9). The possible reasons are in the differences in socioeconomic status and educational level. For instance, only approximately one-fifth of the participants in our study had attained a tertiary education, whereas nearly three-quarters of the participants in the Addis Ababa study had reached this level. Educational status was identified as one of the significant variables associated with the practice of PCC. Study participants who were attending college or university were 3.52 times more likely to undertake PCC. This finding aligns with studies conducted in Wolkite (30), Mizan-Aman (23), Belgium (31), and China (9). This trend indicates that more educated women have better access to information and understand the importance of PCC, which promotes service utilization (24). Although improving educational opportunities for women is a long-term effort, it may have a significant impact on improving the utilization of such services.

Participants in the present study who had previous adverse pregnancy outcomes were 4.82 times more likely to adhere to good practices of PCC, which is similarly reflected in studies conducted in Hosanna (15), Addis Ababa (27), and Debre Tabor (32). This adherence may be attributed to the fear of recurrence of complications among women who had experienced previous adverse pregnancy outcomes. Motivated by a desire to prevent future problems, these women were more likely to attend the counseling provided by healthcare providers and follow their medical advice in subsequent pregnancies.

Study participants who had spousal support were 2.45 times more likely to observe good PCC practices, similar to prior study findings in Fiche (24), Mekelle (18), and China (9). Male engagement is an important strategy for improving maternal health, as it empowers their partners in healthcare decision-making and care-seeking behaviors, including PCC (33).

In this study, participants with a good knowledge of PCC were 4.52 times more likely to practice it effectively. This finding is consistent with studies conducted in Wolkite (30), Mizan-Aman (23), Debre Birhan (17), Debre Tabor (32), Mekelle (18), and Nigeria (21). A current qualitative study also supports this result, highlighting poor awareness of PCC as a key factor affecting its uptake, an issue similarly reported in studies from Jimma (34) and the Netherlands (35). One possible explanation is that women with greater knowledge of PCC are more likely to understand its benefits and the consequences of not utilizing the service. Therefore, increasing awareness among women of PCC may lead to improved practices.

In addition, cultural influences were reported as barriers to the practice of PCC. The majority of women kept their pregnancy intentions secret from others, including their partners and families, as discussing the desire to become pregnant or acknowledging a pregnancy is considered shameful until the possibility of an abortion or miscarriage is ruled out. This finding aligns with studies conducted in Jimma (34), Zimbabwe (36), the Netherlands (35), and Australia (37).

This study also identified transportation costs and service charges as significant barriers to accessing PCC. These financial constraints were similarly reported in studies conducted in Jimma (34) and Zimbabwe (36), highlighting a widespread challenge that undermines equitable healthcare access. As PCC services are not free, and long distances impede access to these services, the majority of women from low-income households may be unable to afford them.

## Limitations of the study

This study addressed the factors that influence the practice of PCC quantitatively and qualitatively among Ethiopian women; however, it could there are limitations associated with this article. First, the cause-and-effect relationship was not shown due to this study's cross-sectional nature. In addition, there is a risk of recall and social desirability biases, which may have affected the accuracy of reported PCC practices, potentially conflated prevalence estimates, and distorted associations. To minimize these effects, interviews were conducted privately, with assurances of confidentiality, and data collectors were trained to use non-leading questions. Another limitation of this study is its broad definition of good practice, which was based on receiving at least one PCC component. This threshold likely overestimated comprehensive PCC coverage, with the 31.3% prevalence reflecting any exposure to PCC, rather than full or recommended care. Although this approach was necessary given the limited availability of services, it may weaken the association with outcomes.

Finally, data saturation was reached after conducting eight in-depth interviews in the qualitative part of the study; however, the authors acknowledge that the sample size is relatively small. This limited number of participants may constrain the transferability of the findings to the broader population of pregnant women.

## Conclusion

This study found that less than one-third of the participating women engaged in good PCC practices; on the other hand, nearly 7 in 10 women became pregnant without engaging in PCC. Attending college or university, having a history of previous adverse pregnancy outcomes, receiving support from a spouse, and having good knowledge of PCC were significantly positively associated with good PCC practices. This qualitative study identified two themes: client-related factors (lack of awareness of the service, economic factors, time constraints, and cultural factors) and health facility factors (not announcing the presence of the service, unavailability of the service at the nearest health facilities, longer waiting times, and service charges) were both identified as reasons for poor practice of PCC.

To improve PCC practices, the Ethiopian Ministry of Health should prioritize expanding health insurance coverage for low-income women to improve accessibility and utilization of PCC services. In addition, increasing awareness about PCC among all reproductive-age women should be emphasized to encourage greater engagement with PCC services. Future studies are encouraged to use a more tiered approach to comprehensive PCC, using terms like “some practice” or “minimal engagement” to more accurately reflect the observed level of care and including PCC utilization among husbands.

## Data Availability

The raw data supporting the conclusions of this article will be made available by the authors, without undue reservation.
